# Engineering strigolactone signaling: toward crops that resist parasites without sacrificing symbiosis

**DOI:** 10.1007/s44297-025-00053-4

**Published:** 2025-06-24

**Authors:** Zeming Huang, Fan Qi

**Affiliations:** 1 Institute of Biotechnology, Zhejiang University, Hangzhou, 310058 China; 2 Xianghu Laboratory, Hangzhou, 311231 China; 3 The Sainsbury Laboratory, Norwich Research Park, Norwich, NR4 7UH UK

Strigolactones (SLs) constitute a class of carotenoid-derived phytohormones that orchestrate developmental plasticity and adaptive responses to environmental cues in plants [1]. The biosynthetic cascade of SLs is initiated by the stereoselective isomerization of all-*trans*-β-carotene to 9-*cis*-β-carotene, which is catalyzed by the isomerase DWARF27 (D27). This cis-configured intermediate undergoes sequential oxidative cleavage: first, carotenoid cleavage dioxygenase 7 (CCD7) catalyzes the stereo-specific cleavage of 9-*cis*-β-carotene to generate 9-*cis*-β-apo-10’-carotenal, which is subsequently remodeled by CCD8 to yield carlactone (CL), the central precursor of SLs [2]. The committed step in SL diversification involves cytochrome P450-mediated oxidation. Members of the cytochrome P450 family 711 subfamily A (CYP711A) oxidize CL to produce carlactonoic acid (CLA), a pivotal branch-point metabolite [2]. Divergent evolutionary trajectories within the P450 superfamily govern subsequent modifications. In *Marchantia paleacea*, the cytochrome P450 enzyme MORE AXILLARY GROWTH 1 (MAX1) catalyzes the conversion of CLA to bryosymbiol (BSB) [3], while in *Vigna unguiculata* and *Solanum lycopersicum*, enzymes from the CYP722C subfamily catalyze the conversion of CLA to the canonical SL orobanchol. The CYP712G1 enzyme then converts orobanchol to solanacol, the predominant SL in tomato root exudates [4]. Furthermore, some CYP722C-subfamily enzymes mediate the conversion of CLA to the strigol-type SL 5-deoxystrigol (5DS). In *Sorghum bicolor*, CYP728B35 subsequently converts 5DS to sorgomol [5]. Legumes may employ distinct enzymes to convert CLA into non-canonical SLs, including lotuslactone and medical compounds [6] (Fig. 1A).

**Fig. 1:**
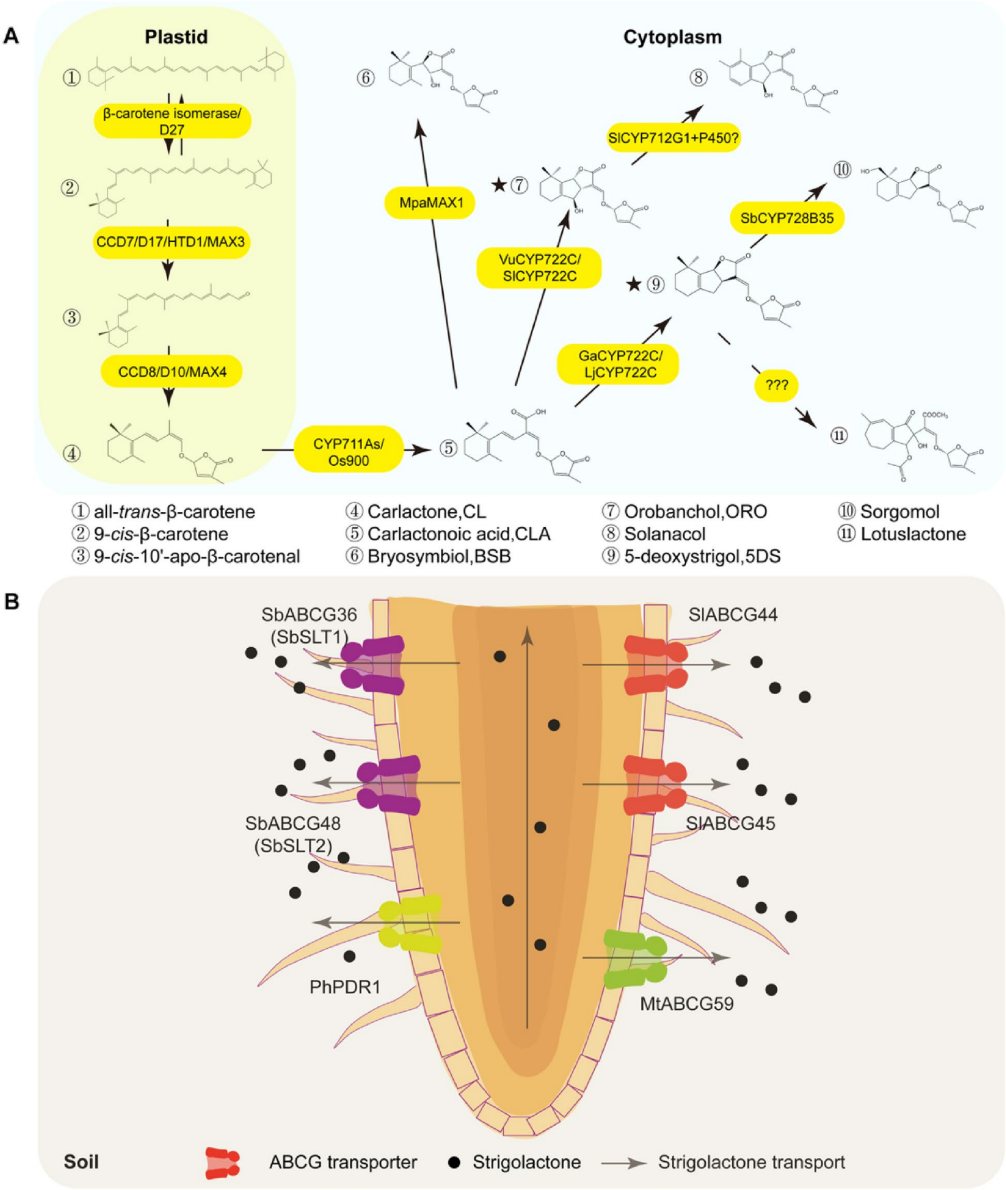
Schematic diagram of strigolactones (SLs) synthetic and transport pathways. **A** Summary of SLs biosynthesis pathways among different plants. SLs molecules are numbered with serial identifiers, and enzymes involved in SLs biosynthesis are highlighted with a yellow background. Canonical SLs are marked with asterisks. **B** Summary of the molecular mechanisms of SLs transport in different plants and fields. Abbreviations: *Os, Oryza sativa; Vu, Vigna unguiculata; Sl, Solanum lycopersicum; Sb, Sorghum bicolor; Lj, Lotus japonicus; Mt, Medicago truncatula;Mpa, Marchantia paleacea; Ga, Gossypium arboreum; Ph, Petunia hybrida*. Enzyme ID: D27, C7AU21; CCD7、MAX3, Q7XJM2; CCD8、MAX4, Q8VY26; CYP711A2、Os900, M9R6D3; MpaMAX1, A0A8D4XJ04; VuCYP722C, Vigun03g264300; SlCYP722C, A0A3Q7F9H0; GaCYP722C, LC528626; LjCYP722C, A0A6F8PJQ1; SlCYP712G1, A0A3Q7IDV9; SbCYP728B35, Sb08g017540

The co-option of SL signaling by parasitic organisms is a byproduct of the synergistic co-evolution between plants and arbuscular mycorrhizal (AM) symbiosis. In the ancient bryophyte *M. paleacea*, secreted SLs, particularly BSB, function as phytohormones that regulate growth and development while also serving as rhizospheric signaling molecules that induce AM fungal symbiosis [3]. Recently, Wang’s group reported that *M. paleacea ccd8a/8b* mutants, which are deficient in SLs, presented significantly impaired AM colonization. This impairment could be partially rescued by exogenous application of GR24 (a synthetic SL analog) or root exudates from phosphorus-deficient plants, which contain endogenous SLs [7].

Divergent evolution in vascular plants has led to the structural diversification of SLs. Monocots, such as sorghum, predominantly secrete non-hydroxylated 5DS to optimize AM symbiosis under low-phosphorus conditions. However, this strategy inadvertently enhances susceptibility to *Striga* [8]. In contrast, plants within the Fabaceae family preferentially synthesize hydroxylated SLs, including orobanchol and lotuslactone [9]. This specialized profile of SLs diminishes the recognition of *Striga* while maintaining effective AM symbiosis, thereby representing an evolutionary adaptation that balances symbiotic benefits and parasitic defenses (Fig. 2).

**Fig. 2:**
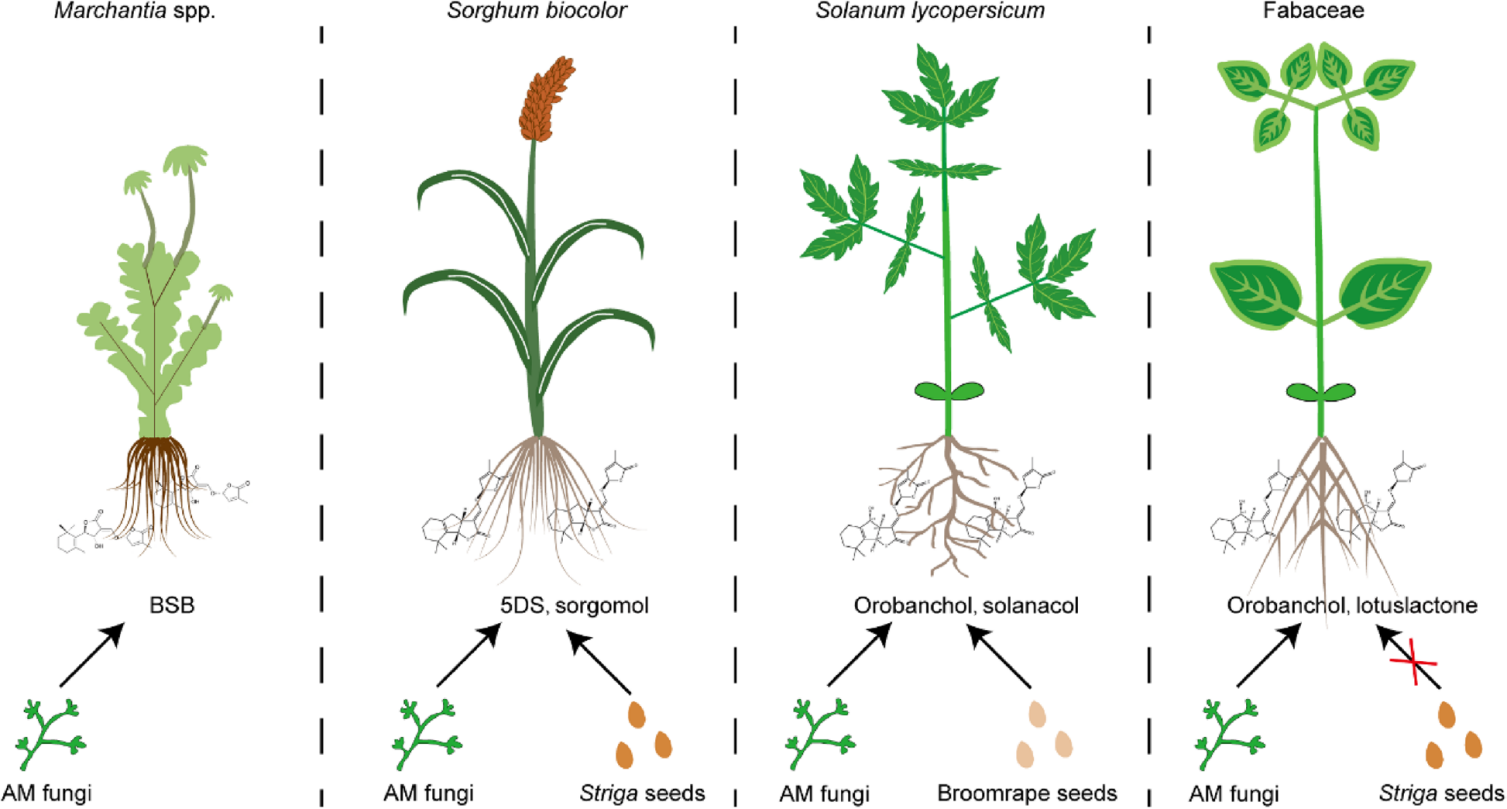
Proposed models of strigolactone (SL) signaling mechanisms in *Marchantia paleacea*, *Sorghum bicolor*, *Solanum lycopersicum* and Fabaceae. In *M. paleacea*, root-excreted SLs predominantly consist of bryosymbols, which function as rhizosphere signaling molecules to promote arbuscular mycorrhizal (AM) fungal symbiosis [3]. *S. bicolor* primarily secretes 5DS under low-phosphorus conditions. While this SL enhances AM fungal colonization, it is paradoxically exploited by parasitic *Striga* seeds to stimulate germination [10]. *S. lycopersicum* primarily secretes orobanchol and solanacol under low-phosphorus conditions. While this SL enhances AM fungal colonization, it is paradoxically exploited by parasitic broomrape seeds to stimulate germination [11]. Fabaceae species predominantly produce orobanchol and noncanonical SLs through root exudation, and these compounds effectively promote AM fungal symbiosis while reducing susceptibility to parasitic plant exploitation [6]. Abbreviations: SL, strigolactone; BSB, bryosymbiol; 5DS, 5-deoxystrigol; AM, arbuscular mycorrhizal

This raises critical questions: do hydroxylation modifications in SLs determine their preferential recognition by AM fungi versus parasitic plants? How can SL transporter-targeted crop systems be engineered to spatially restrict SL secretion (e.g., root tip-specific expression) for precise suppression of *Striga* germination while preserving AM symbiosis in nutrient-deprived zones?

In addition to the biosynthesis of SLs, their secretion into the rhizosphere is pivotal for inducing AM symbiosis and the germination of parasitic plant seeds. In 2012, the ABC transporter G (ABCG) gene *PLEIOTROPIC DRUG RESISTANCE 1* (*PDR1*) in *Petunia hybrida* was identified as the first SL transporter gene [12]. The functions of increasing numbers of SLs transporters, such as those in *Medicago truncatula* [13]**,** *S. bicolor* [10] and *S. lycopersicum* [11], are being progressively elucidated (Fig. 1B).

In a breakthrough study, Shi et al. [10] identified two ABCG transporter genes in sorghum (*S. bicolor*), sorghum *SL transporter 1* (*SbSLT1*) and *SL transporter 2* (*SbSLT2*), which are responsible for exporting SLs from roots into the rhizosphere. These transporters were induced under low phosphorus conditions or GR24 treatment, as confirmed by transcriptomic and functional analyses. Using AlphaFold2, researchers have predicted the protein structures of SbSLT1 and SbSLT2 and validated F693 in SbSLT1 and F642 in SbSLT2 as key binding sites for SLs. CRISPR/Cas9-mediated knockout of these genes disrupted SLs efflux into the rhizosphere, leading to 67–94% suppression of *Striga* seed germination and parasitism in sorghum, thereby stabilizing crop yield. In field trials under *Striga*-free conditions, single (*SbSLT1*^*ko*^, *SbSLT2*^*ko*^) and double knockout (*SbSLT1*^*ko*^*SbSLT2*^*ko*^) mutants presented no growth defects compared with wild-type plants. However, in *Striga*-infested fields, the mutants presented fewer parasitic plants and significantly lower yield losses than did the wild type. Notably, the mutants presented greater aboveground biomass (fresh and dry weights) than did the wild type, which was attributed to prolonged leaf greenness and increased tillering. These results demonstrate that targeting SbSLT1/SbSLT2 effectively controls *Striga* parasitism in agricultural settings, suggesting a promising strategy to mitigate crop losses caused by parasitic weeds.

Furthermore, Ban et al. [11] identified the ABCG transporter genes *SlABCG44* and *SlABCG45* in *S. lycopersicum*, which are involved in the exudation and upward translocation of the SLs orobanchol and solanacol within tomato plants. Both pot experiments and field trials consistently demonstrated that single knockout mutants (*Slabcg45*^*ko*^ and *Slabcg44*^*ko*^) presented a significant reduction in parasitic infestation by Egyptian broomrape. Additionally, the yields of the two *Slabcg45*^*ko*^ mutants increased by more than 30% compared with those of the control group, underscoring the substantial potential application of SlABCG45 in the breeding of parasitic-resistant tomato varieties.

These studies reveal the central roles of SL transporters in rhizospheric SLs secretion, providing novel targets for precision editing to confer plant resistance against *Striga* (witchweed) and broomrape. By integrating AI-predicted evolutionarily conserved SLs transporter motifs, this strategy can be extended to major crops through homology-directed CRISPR editing, enabling tissue-specific SLs secretion control to balance parasite defense and symbiotic efficiency while minimizing off-target effects on root microbiome assembly.

The evolutionary arms race between parasitic plants and their hosts has driven co-adaptive innovations in SLs perception systems. Emerging evidence has demonstrated that noncanonical SLs derivatives in leguminous species are highly distinctive, enabling plants to maintain high efficacy in AM symbiosis while simultaneously evading detection by parasitic plant germination receptors. This structural divergence between canonical and noncanonical SLs variants likely constitutes an evolutionary countermeasure against parasitic infestation. Deciphering the molecular mechanisms underlying SLs biosynthesis pathway diversification and spatiotemporal transport regulation will not only resolve long-standing questions about functional trade-offs in plant chemical ecology but also establish a mechanistic framework for engineering SLs-mediated rhizosphere communication in agricultural ecosystems. Such insights could revolutionize crop improvement strategies by leveraging natural evolutionary solutions to optimize symbiotic partnerships while minimizing parasitic vulnerability.

## Data Availability

Not applicable.

## References

[1] Waters MT, Gutjahr C, Bennett T, Nelson DC. Strigolactone signaling and evolution. Annu Rev Plant Biol. 2017;68:291–322. 10.1146/annurev-arplant-042916-040925. 28125281 10.1146/annurev-arplant-042916-040925

[2] Mashiguchi K, Seto Y, Yamaguchi S. Strigolactone biosynthesis, transport and perception. Plant J. 2021;105:335–50. 10.1111/tpj.15059. 33118266 10.1111/tpj.15059

[3] Kodama K, Rich MK, Yoda A, Shimazaki S, Xie XN, Akiyama K, et al. An ancestral function of strigolactones as symbiotic rhizosphere signals. Nat Commun. 2022;13:3974. 10.1038/s41467-022-31708-3. 35803942 10.1038/s41467-022-31708-3 PMC9270392

[4] Wang Y, Durairaj J, Suarez Duran HG, van Velzen R, Flokova K, Liao CY, et al. The tomato cytochrome P450 CYP712G1 catalyses the double oxidation of orobanchol en route to the rhizosphere signalling strigolactone, solanacol. New Phytol. 2022;235:1884–99. 10.1111/nph.18272. 35612785 10.1111/nph.18272 PMC9542622

[5] Wakabayashi T, Ishiwa S, Shida K, Motonami N, Suzuki H, Takikawa H, et al. Identification and characterization of sorgomol synthase in sorghum strigolactone biosynthesis. Plant Physiol. 2021;185:902–13. 10.1093/plphys/kiaa113. 33793911 10.1093/plphys/kiaa113 PMC8133691

[6] Xie X, Mori N, Yoneyama K, Nomura T, Uchida K, Yoneyama K, et al. Lotuslactone, a non-canonical strigolactone from *Lotus japonicus*. Phytochemistry. 2019;157:200–5. 10.1016/j.phytochem.2018.10.034. 30439621 10.1016/j.phytochem.2018.10.034

[7] Tan X, Wang D, Zhang X, Zheng S, Jia X, Liu H, et al. A pair of LysM receptors mediates symbiosis and immunity discrimination in *Marchantia*. Cell. 2025;188:1330–48. 10.1016/j.cell.2024.12.024. 39855200 10.1016/j.cell.2024.12.024

[8] Chen J, Zhang LM, Zhu MJ, Han LJ, Lv Y, Liu YS, et al. *Non-dormant Axillary Bud 1* regulates axillary bud outgrowth in sorghum. J Integr Plant Biol. 2018;60:938–55. 10.1111/jipb.12665. 29740955 10.1111/jipb.12665

[9] Yoneyama K, Xie XN, Sekimoto H, Takeuchi Y, Ogasawara S, Akiyama K, et al. Strigolactones, host recognition signals for root parasitic plants and arbuscular mycorrhizal fungi, from Fabaceae plants. New Phytol. 2008;179:484–94. 10.1111/j.1469-8137.2008.02462.x. 19086293 10.1111/j.1469-8137.2008.02462.x

[10] Shi J, Mei C, Ge F, Hu Q, Ban X, Xia R, et al. Resistance to *Striga* parasitism through reduction of strigolactone exudation. Cell. 2025;188:1955–66. 10.1016/j.cell.2025.01.022. 39947180 10.1016/j.cell.2025.01.022

[11] Ban X, Qin L, Yan J, Wu J, Li Q, Su X, et al. Manipulation of a strigolactone transporter in tomato confers resistance to the parasitic weed broomrape. The Innovation. 2025;6: 100815. 10.1016/j.xinn.2025.100815. 40098680 10.1016/j.xinn.2025.100815 PMC11910882

[12] Kretzschmar T, Kohlen W, Sasse J, Borghi L, Schlegel M, Bachelier JB, et al. A petunia ABC protein controls strigolactone-dependent symbiotic signalling and branching. Nature. 2012;483:341–4. 10.1038/nature10873. 22398443 10.1038/nature10873

[13] Banasiak J, Borghi L, Stec N, Martinoia E, Jasinski M. The full-size ABCG transporter of *Medicago truncatula* is involved in strigolactone secretion, affecting arbuscular mycorrhiza. Front Plant Sci. 2020;11:18. 10.3389/fpls.2020.00018. 32117367 10.3389/fpls.2020.00018 PMC7019051

